# Transcriptional Profiling of Aging in Human Muscle Reveals a Common Aging Signature

**DOI:** 10.1371/journal.pgen.0020115

**Published:** 2006-07-21

**Authors:** Jacob M Zahn, Rebecca Sonu, Hannes Vogel, Emily Crane, Krystyna Mazan-Mamczarz, Ralph Rabkin, Ronald W Davis, Kevin G Becker, Art B Owen, Stuart K Kim

**Affiliations:** 1 Department of Developmental Biology, Stanford University Medical Center, Stanford, California, United States of America; 2 Department of Pathology, Stanford University Medical Center, Stanford, California, United States of America; 3 Laboratory of Cellular and Molecular Biology, National Institute on Aging, National Institutes of Health, Baltimore, Maryland, United States of America; 4 Department of Medicine, Stanford University Medical Center, Stanford, California, United States of America; 5 Veterans Affairs Palo Alto Health Care System, Palo Alto, California, United States of America; 6 Department of Genetics, Stanford University Medical Center, Stanford, California, United States of America; 7 Department of Biochemistry, Stanford University Medical Center, Stanford, California, United States of America; 8 Research Resources Branch, National Institute on Aging, National Institutes of Health, Baltimore, Maryland, United States of America; 9 Department of Statistics, Stanford University, Stanford, California, United States of America; The Jackson Laboratory, United States of America

## Abstract

We analyzed expression of 81 normal muscle samples from humans of varying ages, and have identified a molecular profile for aging consisting of 250 age-regulated genes. This molecular profile correlates not only with chronological age but also with a measure of physiological age. We compared the transcriptional profile of muscle aging to previous transcriptional profiles of aging in the kidney and the brain, and found a common signature for aging in these diverse human tissues. The common aging signature consists of six genetic pathways; four pathways increase expression with age (genes in the extracellular matrix, genes involved in cell growth, genes encoding factors involved in complement activation, and genes encoding components of the cytosolic ribosome), while two pathways decrease expression with age (genes involved in chloride transport and genes encoding subunits of the mitochondrial electron transport chain). We also compared transcriptional profiles of aging in humans to those of the mouse and fly, and found that the electron transport chain pathway decreases expression with age in all three organisms, suggesting that this may be a public marker for aging across species.

## Synopsis

Aging is a complex phenomenon characterized by the decay of biological function over time, eventually leading to death. High-throughput methods for examining changes in the expression of genes, such as DNA microarrays, have been successful in elucidating some of the genome-wide changes that occur with age in several human tissues. The authors profiled gene expression changes in the muscles of 81 individuals with ages spanning eight decades. They found 250 genes and 3 genetic pathways that displayed altered levels of expression in the elderly. The transcriptional profile of age-regulated genes was able to discern elderly patients with severe muscle aging from those that retained high levels of muscle function; that is, the gene expression profiles reflected physiological as well as chronological age. In order to find genetic changes that might affect most or all tissues during aging, the authors compared genome-wide profiles of aging in the muscle to those in the kidney and the brain, and found a common signature for aging shared among these three tissues consisting of six genetic pathways. One of these aging pathways (the electron transport chain pathway) is age regulated not only in humans but also in two model organisms (mice and flies), providing insights about shared age-related changes in animals with vastly different lifespans.

## Introduction

Aging is marked by the gradual decline of a multitude of physiological functions leading to an increasing probability of death. Some aging-related changes affect one's appearance, such as wrinkled skin, whereas others affect organ function, such as decreased kidney filtration rate and decreased muscular strength. At the molecular level, we are just beginning to assemble protein and gene expression changes that can be used as markers for aging. Rather than search for molecular aging markers by focusing on only one gene or pathway at a time, an attractive approach is to screen all genetic pathways in parallel for age-related changes by using full-genome oligonucleotide chips to search for gene expression changes in the elderly. A genome-wide transcriptional profile of aging may identify molecular markers of the aging process, and would provide insight into the molecular mechanisms that ultimately limit human lifespan.

Molecular markers of aging must reflect physiological function rather than simple chronological age because individuals age at different rates [1]. In the mouse, changes in the levels of CD4 immunocytes and changes in the expression of cell-cycle genes such as *p16^INK4a^* are molecular markers of aging, as they predict both the remaining lifespan and the physiological age of the mouse [2–4]. In the human, gene expression profiling experiments identified 447 age-regulated genes that could predict the physiological age of the kidney [5]. Whole-genome expression profiling has also been used to identify genes that change expression with chronological age in the brain [6], skeletal muscle [7,8], and dermal fibroblasts [9], but changes in expression of these marker genes have not yet been shown to correlate with physiological aging.

In this paper, we have performed a genome wide analysis of gene expression changes in the human skeletal muscle. As age increases, skeletal muscle degenerates, loses mass, loses total aerobic capacity, and becomes markedly weaker [10]. One measure of muscle physiology is the ratio of the diameters of the type I and type II muscle fibers. A decrease in the size of type II muscle fibers (fast twitch) has been found to be correlated with decline in muscle function in both human [11] and rat [12]. Type II muscle fibers are known to atrophy and become smaller with age in the human, partially accounting for decreased muscle strength and flexibility in old age. As type II muscle fibers become smaller with age, the ratio of the diameters of type II fibers to type I fibers becomes smaller.

The extent to which age regulation of genetic pathways is specific to a particular tissue or common across many tissues is unknown. Age regulation of gene expression between the cortex and medulla regions of the human kidney was found to be highly correlated [5]. There was a high correlation in gene expression changes with age in different regions of the brain cortex, but no similarity was found between the cortex and the cerebellum [13]. Thus, there are similarities in patterns of age regulation between different areas of the kidney and between different areas of the brain cortex, but a common signature for aging across many diverse tissues has not been found.

Another key issue is whether there are genetic pathways that are commonly age regulated in different species with vastly different lifespans, such as human, mouse, fly, and worm. Transcriptional profiles of aging have been performed on both skeletal muscle and brains in the mouse [14,15], in Drosophila melanogaster [16,17], and in Caenorhabditis elegans [18]. A comparison of the patterns of gene expression changes during aging in the fly and the worm concluded that genes encoding mitochondrial components decreased expression with age in both species [19].

In this work, we present a transcriptional expression profile of 81 human skeletal muscle samples as a function of age. The symporter activity, sialyltransferase activity, and chloride transport pathways all decrease expression with age in human muscle. The age-regulated genes were found to be markers of physiological age, not just chronological age. By comparing our results on aging in muscle to previous transcriptional profiles of aging in the kidney and the brain, we found a common signature for aging across different human tissues consisting of six genetic pathways that showed common patterns of age regulation in all three tissues. Finally, by comparing the signature for aging in humans to transcriptional profiles of aging in mice, flies, and worms, we found that expression of the electron transport chain decreases with age in humans, mice, and flies, constituting a public signature for aging across species with extremely different lifespans.

## Results

### A Global Gene Expression Profile for Aging in Human Muscle

In order to study the effects of aging in human muscle, we obtained 81 samples of human skeletal muscle from individuals spanning 16 to 89 y of age (Table 1). Sixty-three samples were obtained from the abdomen, 5 were obtained from the arm, 2 were obtained from the deltoid muscle, 2 were obtained from the inner thigh, and 9 were obtained from the quadriceps (Table S1). We used Affymetrix DNA arrays to generate a transcriptional profile of aging in human muscle. We isolated total RNA from each muscle sample, and synthesized biotinylated cRNA from total RNA. We then hybridized the cRNA to Affymetrix 133 2.0 Plus oligonucleotide arrays, representing nearly the entire human genome (54,675 individual probe sets corresponding to 31,948 individual human genes). We plotted the expression of each gene as a function of age, resulting in a dataset that shows the expression of nearly every gene in the genome as a function of age in human muscle (data are publicly available on the Gene Expression Omnibus at http://www.ncbi.nlm.nih.gov/geo).

**Table 1:**
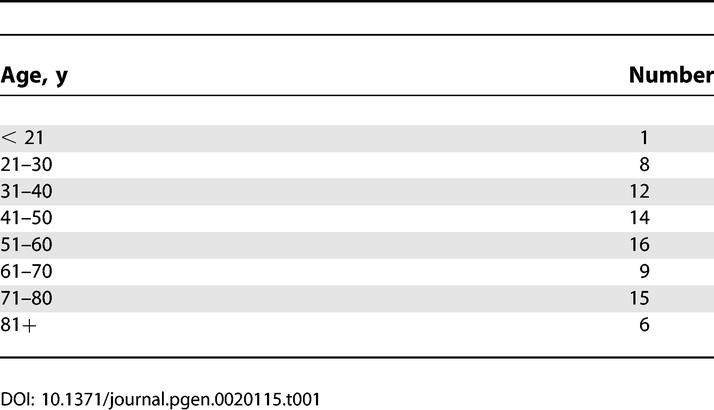
Patients Recruited by Age Group

We used a multiple regression technique on each gene to determine how its expression changes with age, as had been done previously for age regulation in the kidney (Materials and Methods) [5]. We analyzed age regulation in skeletal muscle in two ways. In the first way, we found individual genes that met a stringent statistical significance threshold for correlation with age. In the second way, we found groups of genes (defined by the Gene Ontology consortium) in which there is subtle but consistent age regulation.

**Figure 1:**
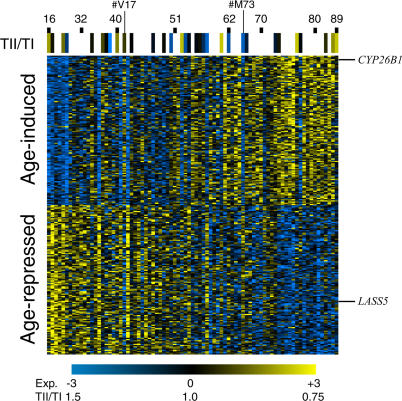
Expression of 250 Age-Regulated Genes in Muscle Rows correspond to individual genes, arranged in order from greatest increase in expression with age at top to greatest decrease in expression with age at bottom. Columns represent individual patients, from youngest at left to oldest at right. Ages of certain individuals are marked for reference. Scale represents log_2_ expression level (Exp). Genes discussed in the text are marked for reference. A navigable version of this figure showing identities of specific genes can be found at http://cmgm.stanford.edu/~kimlab/aging_muscle.

To identify individual genes showing strong age regulation, we examined the slope with respect to age for each gene, and identified 250 genes in which the slope was significantly positive or negative (*p* < 0.001) (Figure 1, Table S2, and Materials and Methods). At this statistical threshold, we would expect only 32 genes by chance, suggesting a false discovery rate of 13% or less. Furthermore, we randomly permuted the ages of the muscle samples, keeping the gene expression, sex, and anatomy variables fixed, and counted the number of genes that were significantly age regulated, again at *p* < 0.001. In 1,000 such permutations we found fewer than 107 significant genes 95% of the time. Thus, we are confident that most of the 250 age-regulated genes are not sampling artifacts. Of the 250 age-regulated genes, 125 genes increase expression, and 125 genes decrease expression with age.

We considered the possibility that some of the 250 genes might not be age regulated per se, but rather might appear to be age regulated because they are associated with a pathological condition that increases with age. For example, the incidence of diabetes is known to increase with age in the general human population [20]. Our selection of patients might show a bias of diabetes in the elderly, in which case genes that change expression in response to diabetes might appear to be age regulated in our study. In addition to diabetes, we considered thirteen other factors that might also confound our study on aging, such as whether the patient was male or female, the anatomical origin of the muscle sample, the type of pathology associated with the patient, and types of medication taken by the patient (Table S1).

With the exception of hypothyroidism, none of the medical factors showed a strong association with age, and so it is unlikely that these confounding factors would cause genes to appear to be age regulated (Figure S1). Hypothyroidism was absent in the young and present in about half of the elderly.

We used two methods to test whether any of the factors affected the slope of gene expression with respect to age of the 250 age-regulated genes. First, we used a multiple regression model that included a fourth term representing the medical factor (such as hypothyroidism) in addition to age, sex, and anatomy. We then compared the aging coefficient using this new model with the one from the original model that did not include the term. If any of the 250 genes were regulated by the medical factor and not by age per se, we would expect marked differences in the aging coefficients generated by the two multiple regression models. None of the fourteen medical factors, including hypothyroidism, had a significant effect on age regulation (Figure S2). Second, we performed an unsupervised hierarchical cluster analysis of the 250 age-regulated genes. If our analysis of age regulation were confounded by a medical factor, we would expect that the presence of the medical factor would be clustered when we sorted the 81 patients according to their patterns of gene expression. None of the pathological or pharmaceutical factors showed clustering (Figure S3). Most of the nonabdominal samples were from young patients, and there was some clustering of the muscle samples according to their anatomical origin as expected. This clustering does not affect our analysis of age regulation because anatomical origin was included as a term in the multiple regression model. Thus, these two methods showed no evidence that anatomical, pathological, or pharmaceutical factors confound the results of our aging study.

In summary, we have generated a global profile of changes in gene expression during aging in human muscle (Figure 1). It is well established that aging has many effects on muscle, such as decrease in physiological performance, changes in morphology, and increased susceptibility to disease. The data from Figure 1 extend our understanding of muscle aging to the level of specific genes and genetic pathways, providing insight into possible mechanisms underlying overall decline of muscle function in old age. Overall, the difference in gene expression between young and old muscle tissue is relatively small. Specifically, only 250 genes show significant changes in expression with age (*p* < 0.001), and the large majority of these age-regulated genes change expression less than two-fold in 50 y. These results are consistent with a model in which age-related decline in cellular functions is caused by the accumulation of multiple, minute changes in the regulation of genes and pathways.

The genetic functions of many of the 250 genes shown in Figure 1 are known, and some suggest biological mechanisms that could cause age-related decline in muscle physiology. For example, *CYP26B1* shows an average increase in expression of 90% in 50 y. *CYP26B1* is a member of the cytochrome P450 family, which are monoxygenases used to metabolize toxic substances. Increased expression of *CYP26B1* in old age could help eliminate toxins that accumulate with age.

*LASS5* decreases expression approximately 25% in 50 y. *LASS5* is the human ortholog of the yeast *lag1* longevity assurance gene. In yeast, *lag1* expression decreases in older yeast cells [21] similar to our results showing decreased expression in old age in human muscle. *LASS5* is involved in the ceramide signaling pathway, which plays important roles on several lifespan-associated processes, such as stress resistance and apoptosis [22]. Reduced expression of *LASS5* in old age could impair cell function by reducing ceramide signaling.

In addition to searching for age regulation one gene at a time, we also screened known genetic pathways for those showing an overall change with age. With this approach, age regulation for every gene in a pathway is combined to determine whether there is an overall regulation of the entire pathway. Screening for coordinated age regulation of genetic pathways increases the sensitivity of our analysis, as the combined effects of small regulation of many genes in a pathway can be significant. For example, in a previous study of type 2 diabetes, screening genetic pathways for changes in expression provided key insights that were not possible from analyzing genes individually [23].

We developed a variant of gene set enrichment analysis (GSEA) to determine whether a genetic pathway shows evidence for age regulation [23]. We assayed 624 gene sets defined by the Gene Ontology consortium [24] (Table S3). We modified the original GSEA paradigm because it was intended for datasets with two categories of sample, and we were instead fitting regression models to continuously varying independent and dependent variables. Accordingly, we replaced the two-sample test statistic in GSEA with an estimated regression slope for age. We also replaced the Kolmogorov-Smirnov statistic with a van der Waerden statistic because we prefer the type of dependence that the van der Waerden statistic captures. Finally, we replaced the permutation strategy with a bootstrap in order to better handle covariates (Materials and Methods).

**Figure 2:**
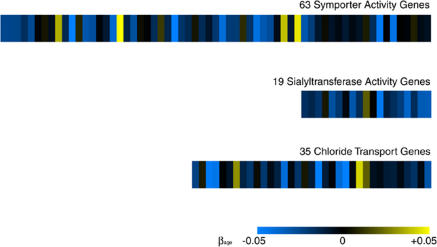
Three Gene Sets Are Regulated with Age in Muscle Rows represent the symporter activity, sialyltransferase activity, and chloride transport gene sets. Columns correspond to individual genes within a given gene set. Scale represents the slope of the change in log_2_ expression level with age (*β_1j_*). A navigable version of this figure showing identities of specific genes can be found at http://cmgm.stanford.edu/~kimlab/aging_muscle.

Our version of the GSEA algorithm scores a gene set according to how the genes in it show coordinated increase (or decrease) on average in response to increasing age. The increase is measured by a van der Waerden statistic. To judge whether a specific van der Waerden statistic is significant, we used bootstrap resampling. Each bootstrap sample was drawn by resampling the arrays and keeping the gene expression measurements linked with the age, sex, and anatomy variables. The 624 van der Waerden scores for the gene groups were recomputed for each of the 1,000 bootstrap samples. Six gene sets were found to have statistically significant van der Waerden scores (*p* < 0.001) in this resampling. At this *p* value threshold, we would only expect to find 0.6 gene sets by chance alone. We also required the gene groups to show some practical significance by rejecting groups with a van der Waerden score smaller than 3.1 in absolute value. We found three pathways that passed both criteria: symporter genes, sialyltransferases, and chloride transporters showed decreasing expression with age (Figure 2 and Table 2). Aging coefficients for all genes in these pathways are listed in Table S4.

**Table 2:**
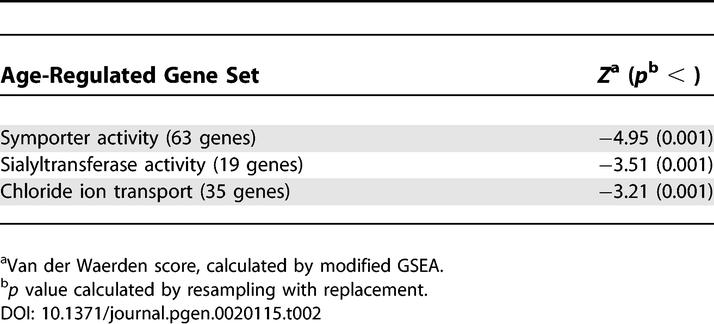
Age-Regulated Gene Sets in Muscle

Symporter genes (63 genes) and chloride transporters (35 genes) are necessary for transporting solutes during muscle contraction [25]; the decreased expression levels of these transporters may be associated with weakness of old muscle. Genes with sialyltransferase activity (19 genes) mediate glycosylation by transferring sialic acid groups to secreted molecules. Decreases in sialyltransferase activity have been previously detected in aging human serum [26], neurons [27], and lymphocytes [28].

### Molecular Markers of Physiological Aging

**Figure 3:**
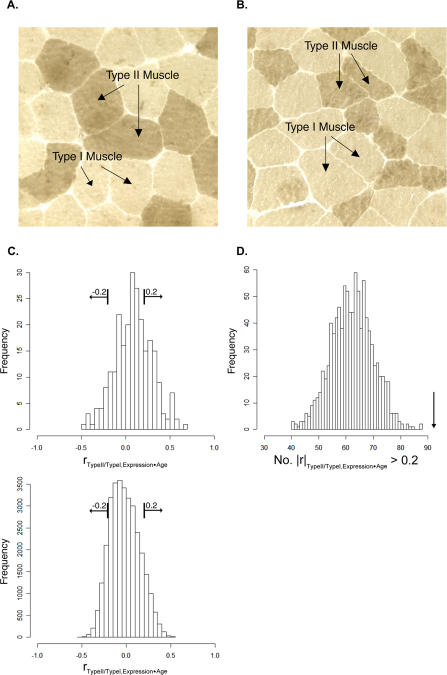
Gene Expression Predicts Physiology of Aging (A) Cross-section of histologically unremarkable deltoid muscle from a 48-y-old woman demonstrating relatively equivalent sizes of types I and II muscle fibers. Arrows denote fibers types as distinguished by enzyme histochemistry (cryosection, 200×, myosin ATPase at pH 9.4). (B) Cross-section of deltoid muscle from an 88-y-old woman demonstrating selective atrophy of type II muscle fibers that stain darkly by ATPase enzyme histochemistry (cryosection, 200×, myosin ATPase at pH 9.4). (C) Histograms showing a correlation between muscle physiology and gene expression for age-regulated genes. Top panel: for each of the 250 age-regulated genes, we calculated the partial correlation coefficients between the type II/type I muscle fiber diameter ratio and gene expression excluding age variation (*x*-axis). Bottom panel: same as top panel, except that correlation coefficients were calculated for all 31,948 genes. The squared partial correlation coefficient denotes the amount that changes in gene expression account for variance in type II/type I muscle fiber diameter ratios while excluding the effects of age. (D) Histogram showing the likelihood of finding 92 genes with |*r*| > 0.2 from a set of random genes. We performed a Monte Carlo experiment by randomly selecting sets of 250 genes from the genome, and calculating how many genes in the set had |*r*| > 0.2 as in (C). The procedure was repeated 1,000 times and the histogram shows the number of genes from each random selection that have |*r*| > 0.2. The arrow shows the number of genes exceeding this threshold (92) from the set of 250 age-regulated genes (*p* < 0.001). We also determined the total number of genes in the genome with |*r*| > 0.2, and then showed that 92 genes from a set of 250 is significant (hypergeometric distribution; *p* < 1 × 10^−4^).

Some people age slowly and remain strong and fit in their 70s, whereas others age rapidly, becoming frail and susceptible to age-related disease. We wanted to determine whether the expression profile for the 250 aging-regulated genes correlated with physiological in addition to chronological aging. For example, patient V17 was 41 y old but expressed his age-regulated genes similarly to patients who were 10 to 20 y older, and we would like to determine whether this patient had poor muscle physiology for his age (Figure 1). Conversely, patient M73 was 64 y old but had a molecular profile similar to other patients that were 30 y younger, and we wanted to determine whether this patient had relatively good muscle physiology for his age. Our list consists of 250 genes that correlate significantly with chronological age. We sought to determine whether they also correlate with physiological age, as measured by the type II/type I diameter ratio. We prepared histological sections for all 81 skeletal muscle samples, and were able to reliably measure the diameters of the type I and type II muscle fibers for 32 samples (Figure 3A and 3B; Table S5).

A simple correlation of gene expression with muscle type ratio would not be sufficient for our purposes. Such a correlation could arise simply because the gene expression and muscle type ratio are both correlated with age. Accordingly, we employed partial correlations of gene expression with muscle type ratios after adjusting for the effect of chronological age. To do this, we regressed type II/type I muscle fiber diameter ratio on age, regressed gene expression on age, and finally correlated residuals from both regressions to obtain partial correlation coefficients. The partial correlations for the 250 age-related genes are shown in Figure 3C.

If a gene correlates with muscle diameter ratio only because both it and muscle diameter are correlated with age, then the partial correlation described above should be close to zero. We found that a large number of the genes in our list had a statistically significant relationship with type II/type I ratio after adjusting for age. However, many of the genes not on our list were also related to type II/type I ratio adjusted for age. We were able to show that genes with large partial correlations were significantly overrepresented in our list of 250 age-regulated genes. We counted 92 of 250 age-related genes for which the (absolute) partial correlation was more than 0.2 (Table S6). There were only 7,768 of 31,948 genes not in the list with a partial correlation this large. Using a hypergeometric distribution, we found a *p* value below 0.0001 and concluded that the age-related genes are more likely than other genes to have some partial correlation with muscle diameter ratio. To illustrate this effect, we also sampled 250 genes from the genome 1,000 different times, each time counting how many had a partial correlation larger than 0.2 in absolute value. None of the samples had a count larger than 92 (Figure 3D).

Our result indicating that the 250 age-regulated genes are enriched for genes regulated by type II/type I muscle fiber diameter ratio is valid even when we use other selection thresholds for muscle physiology (i.e., other than the absolute of *r* > 0.2). We compared the distribution of partial correlations of the 250 age-regulated genes with type II/type I ratios to the distribution of partial correlations of the rest of the genes in the genome using nonparametric methods (Figure 3C). Using a Kolmogorov-Smirnov goodness-of-fit test, we found that the distribution of the 250 age-regulated genes is wider than the total distribution in a two-sided test (*p* < 1 × 10^−15^, with D = 0.27). This result indicates that the apparent physiological basis of our gene set is not a consequence of our having chosen 0.2 as a threshold.

In summary, these statistical tests show that the set of age-regulated genes are markers of the relative level of muscle function, even among patients that are similar in age. Our findings are further supported by two additional statistical tests described in Materials and Methods (Tables S7 and S8). Thus, the age-regulated genes are enriched for those that predict physiological, not just chronological, age. The correlation between gene expression profile and physiological age can be seen in patients V17 and M73 in Figure 1. Although patient V17 is relatively young (41 y old), the gene expression profile for the 250 age-regulated genes is most similar to older individuals, and the type II/type I muscle fiber diameter ratio is low for his age. Conversely, although patient M73 is relatively old (64 y old), the gene expression pattern is similar to younger individuals, and the type II/type I muscle fiber diameter ratio is high for his age (Figure 1).

### A Common Signature for Aging in Muscle, the Kidney, and the Brain

Some aspects of aging affect only specific tissues; examples include progressive weakness of muscle, declining synaptic function in the brain, or decreased filtration rate in the kidney. Other aspects of aging occur in all cells regardless of their tissue type, such as the accumulation of oxidative damage from the mitochondria, DNA damage, and protein damage. Our genome-wide search for gene expression changes during aging would include both types of expression changes, and it would be interesting to discern which expression changes are muscle specific and which are common to all tissues. Expression profiles that are common to aging in all tissues would provide insight into the core mechanisms that underlie cellular aging. Therefore, we compared the DNA chip expression data from our studies on muscle aging to previous DNA chip expression studies on aging in the brain and the kidney. Rodwell et al. have characterized gene expression changes with age in the cortex and the medulla of the kidney from 74 patients, and Lu et al. have examined gene expression changes in the frontal cortex of the brain from 30 patients [5,6].

Our initial attempt to compare transcriptional changes between tissues relied on a Venn analysis, in which we directly compared the overlap in the lists of the age-regulated genes from the three tissues. Next, we searched for a common aging signature by comparing the Pearson correlation of age regulation between two tissues. Both of these straightforward methods showed only borderline statistical evidence for similarities in aging between the three tissues (Materials and Methods), but neither is expected to be powerful. Ultimately, we compared tissues using a grouped gene analysis. Grouping genes can be more powerful if there are small but consistent effects in each of a number of genes. Furthermore, the specific biological processes associated with each genetic pathway provide insights into mechanisms of aging. We used the modified GSEA described above to analyze previously published data on age regulation in the kidney and the brain [5,6]. As before, we considered the possibility that the observed correlations could be due to the fact that there might be random sampling differences in the different tissues that coordinately affect the expression levels of genes in an entire gene set. To control for this possibility, we resampled the microarray data 1,000 times (with replacement) and repeated the analysis of 624 gene sets on every resample. We selected only gene sets that were statistically significant in all three tissues at *p* < 0.05. We then removed any gene set that did not attain a van der Waerden score of 1.65 or more in absolute value in all three tissues. From a total of 624 sets of genes, we found that extracellular matrix genes, cell growth genes, and complement activation genes significantly increase expression with age on average in all three human tissues, whereas chloride transport genes and electron transport genes significantly decrease expression on average with age in those same tissues (Table 3). The cytosolic ribosomal pathway showed increased expression that was significant in the muscle and kidney, and almost significant in the brain (bootstrap *p* < 0.06). Aging coefficients for all genes in each of these pathways are listed in Table S9. We would expect 0.08 (essentially none) of the 624 pathways to appear commonly age regulated by chance (*p* < 0.05 in all three tissues, and hence a combined *p* < 1.25 × 10^−4^).

**Table 3:**
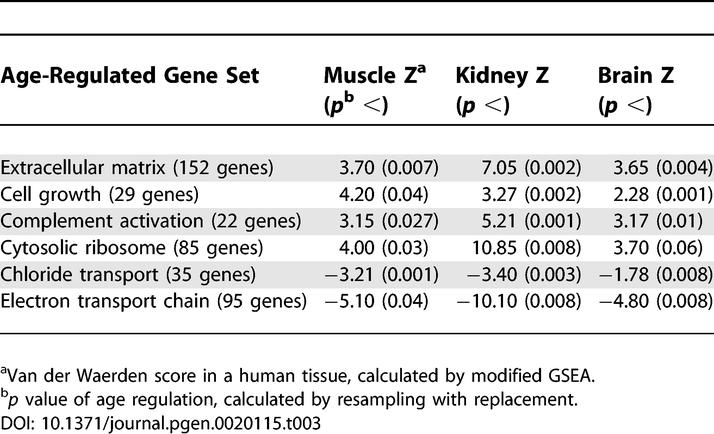
Age Regulation of Gene Sets in Three Human Tissues

**Figure 4:**
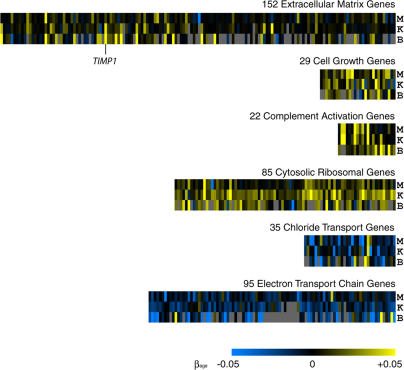
A Common Signature for Aging in Muscle, the Kidney, and the Brain Shown are expression data from sets of extracellular matrix genes, cell growth genes, complement activation genes, cytosolic ribosomal genes, chloride transport genes, and electron transport chain genes. Rows are human tissues (M, muscle; K, kidney; B, brain). Columns correspond to individual genes in each gene set. Scale represents the slope of the change in log_2_ expression level with age *(β_1j_).* Gray indicates genes were not present in the dataset. A navigable version showing identities of specific genes can be found at http://cmgm.stanford.edu/~kimlab/aging_muscle.

Increased overall expression of the extracellular matrix gene set (152 genes) with advancing age may contribute to widespread fibrosis in the elderly (Figure 4). Fibrosis is a process by which fibrous connective tissue proliferates throughout organs and impairs function of many tissues. *TIMP1,* which encodes tissue inhibitor of metalloproteinase 1, shows the largest increase in expression with age (average of 236% in 50 y).

The cell growth gene set (29 genes) includes genes coding for growth factors, such as *TGFB1* and *FGFR1.* Induction of genes in this gene set may reflect an attempt to repair tissue damage that accumulates over lifespan.

Although complement activation genes (22 genes) are induced in muscle, the kidney, and the brain, they are expressed primarily in liver [29]. Therefore, unless complement genes are also age regulated in the liver, the physiological relevance of age regulation of complement genes in muscle, the kidney, and the brain is currently unclear.

Cytosolic ribosomal genes include 85 genes that show a general increase in expression with age in all three tissues. This result is interesting because the rate of protein synthesis is known to decrease in old age [30], and yet our expression results show an increase in the expression of ribosomal genes. One possibility is that decreased protein synthesis in old cells induces expression of ribosomal genes as part of a homeostatic feedback loop to partially compensate for loss of translational efficiency.

The chloride transport pathway is composed of 35 genes that show an overall decrease in expression with age in all three tissues. Ion transport of many types is important not only in the contraction of muscle [25], but also for maintenance of salt balance in the kidney [31] and neuron function in the brain through GABA-mediated receptors [32]. Decreased transport of chloride with age could lead to many types of physiological decline linked to ion transport deficiency.

The mitochondrial electron transport chain was found to show an overall decrease in expression with age. This group contains 95 genes, including genes associated with the NADH dehydrogenase family (complex I), succinate-coenzyme Q reductase (complex II), ubiquinone-cytochrome c reductase (complex III), cytochrome c oxidase (complex IV), H^+^-ATP synthase (complex V), and the uncoupling proteins. The finding that expression of genes involved in the electron transport chain decreases in old age supports the mitochondrial free-radical theory of aging [33], as free-radical generation by mitochondria would preferentially damage the electron transport chain protein complex. Decreased expression of the electron transport genes (encoded in the nucleus) might be caused by feedback regulation from damage to the electron transport chain protein complex. Other protein complexes in the mitochondria (such as mitochondrial ribosomal genes) do not decrease expression with age. Thus, aging does not have a general effect on genes encoding mitochondrial components, but rather specifically affects expression of genes that are part of the electron transport chain.

The above results show that there is common age regulation for these six genetic pathways in the kidney, muscle, and the brain. Next, we determined that there was little statistical evidence for the correlation of age regulation of individual genes in a pathway in one tissue with their age regulation in another tissue (Materials and Methods). Thus, it is unclear whether or not the same genes or different genes within a pathway show age regulation between different tissues. For example, certain genes in the electron transport pathway might be age regulated in the kidney, whereas other electron transport genes might be age regulated in the muscle.

### A Public Age-Regulated Pathway in Humans, Mice, and Flies

Having identified genetic pathways that are commonly age regulated in different human tissues, we next determined whether their age regulation is specific for humans (private) or whether these groups are also age regulated in other species (public). Genetic pathways that are age regulated in different species would be of particular interest because they would identify mechanisms that are inextricably related to aging, even in animals that have vastly different lifespans.

We compared age regulation in humans to previously published studies of age regulation in D. melanogaster [16] and C. elegans [18]*.* To examine age regulation in aging mouse kidneys, we collected a kidney sample from ten C57BL/6 mice at 1, 6, 16, and 24 mo of age for a total of 40 mouse kidney samples. RNA from each kidney was extracted, labeled with P^33^-dCTP, and hybridized to cDNA filter membranes comprising 16,896 cDNA clones corresponding to 11,512 unique genes. We normalized expression values using the Z-score method [34], and analyzed age regulation of each gene using a multiple regression model taking into account age and sex of each mouse donor. Table S10 shows the slope of expression with respect to age for each gene.

We first identified orthologs of human genes in each of the other three species. Next, we determined the change in expression with respect to age for each gene in each species, using multiple regression techniques similar to the ones used for our studies of aging in human muscle (Material and Methods). We took the six gene sets shown to be aging-regulated in diverse human tissues, and then asked whether they also showed age regulation in any of the other three species. We analyzed the expression of each of the gene sets using modified GSEA to determine whether they showed an overall bias in expression with age in each species. Extracellular matrix genes, cell growth genes, complement activation genes, cytosolic ribosomal genes, and chloride transport genes did not show age regulation in other species.

**Figure 5:**
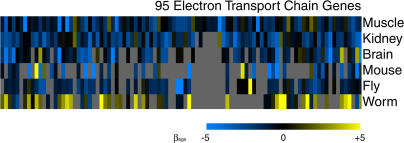
The Electron Transport Chain Decreases Expression with Age in Humans, Mice, and Flies Rows represent either human tissues or model organisms. Columns correspond to individual human genes and homologs to human genes defined by reciprocal best BLAST hits in other species. Scale represents the normalized slope of the change in log_2_ expression level with age (*β_1j_*). Data from different species were normalized by dividing the slope of expression with age by the standard deviation of all similar slopes in the dataset. Gray indicates genes were not present in that species. A navigable version of this figure showing identities of specific genes can be found at http://cmgm.stanford.edu/~kimlab/aging_muscle.

The electron transport chain genes showed a consistent overall decrease in expression with age in humans, mice, and *Drosophila,* but did not show significant age regulation in C. elegans (Figure 5 and Table 4). To show that age regulation is not likely to be due to random biological sampling error, we resampled the electron transport data set in each species with replacement and found that the electron transport chain genes showed significant age regulation in mice (*p* < 0.02) and flies (*p* < 0.001) but not *C. elegans.* The electron transport chain gene set also shows a large van der Waerden score in mice and flies (less than −3.7). In summary, humans, mice, and flies show decreased expression of the electron transport chain during aging, defining a public pathway for aging across species with very different lifespans. In *C. elegans,* it is unclear whether the lack of support for age regulation of the electron transport chain pathway is because the pathway is not age regulated or because the DNA microarray experiments lack statistical power to detect age regulation.

**Table 4:**
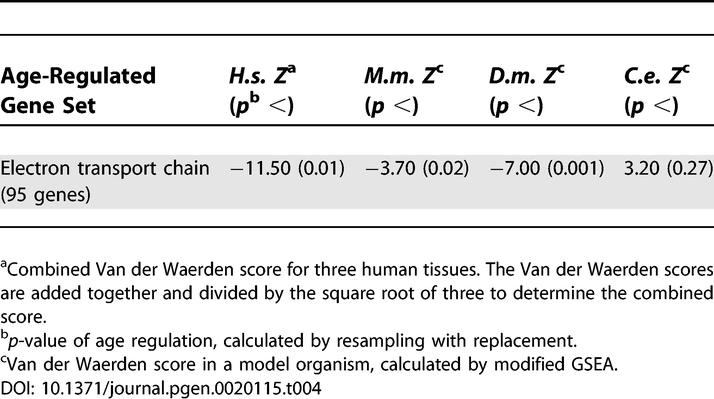
Age Regulation of the Electron Transport Chain in Three Species

## Discussion

In this study, we have generated a high-resolution transcriptional profile of aging in the human muscle. Welle et al. have previously used DNA chips to profile expression changes during aging for the human muscle [7,8], and this work extends their previous studies because we used a significantly larger sample size that enabled much higher statistical resolution.

People age at different rates, especially with regard to muscular aging. Some remain fit and strong, whereas other become frail and weak when they are old. The transcriptional profile for aging in this study reflects the physiological age of the subjects, as measured by muscle diameter ratio, after making an adjustment for their chronological ages. Previous work on age regulation in the kidney also identified molecular markers that could predict the physiological age of the kidney [5].

Our results provide the some of the first evidence for a common signature of changes of gene expression in different human tissues. Specifically, we found similar patterns of age regulation for six biological pathways in the muscle, the kidney, and the brain. Previous studies found similar patterns of aging between different parts of the same tissue, but not between entirely different organs (i.e., age regulation was found to be similar between the cortex and medulla of the kidney [5] and between the frontal pole and the prefrontal cortex in the brain [13]).

Except for the complement activation gene set, the pathways that show common age regulation in diverse tissues also function in all cells. Changes in expression of these pathways in old age may lead to degeneration of not only core cellular functions (such as ion transport and energy production) but also to degeneration of tissue-specific functions (such as kidney filtration and synaptic signaling) that rely on housekeeping pathways. By identifying a common aging signature across tissues, we can now focus on aging pathways that are general instead of tissue-specific. The common aging signature reflects the age of diverse organs, whereas genes that are age regulated in just one tissue reflect the age of that tissue. Finally, treatments or therapies that alter expression of the four common age-regulated pathways might be expected to affect diverse tissues instead of a specific tissue, and may therefore have an overall effect on longevity.

Although some patterns of aging are similar between different human tissues, much of aging is tissue-specific. Decreases in expression of the sialyltransferases and symporter genes are changes specific to muscle, and do not appear to occur in either the kidney or the brain.

Nearly all of the age regulation that we found is specific to humans, and does not seem to occur in old mice, flies, or worms. Thus, much of age regulation in humans is species-specific (private) rather than universal for all animals (public). This result emphasizes the importance of studying aging in humans rather than model organisms with short lifespans in order to understand how people grow old.

Nevertheless, we did find one pathway that was age regulated in humans, mice, and flies. The electron transport chain gene pathway decreases expression with age in all three species. Previous studies found little or no similarity in age regulation between humans and mice [5] or primates [13]. These studies might have overlooked public patterns of age regulation in different species because they searched for similarities in age regulation at the level of individual genes rather than of entire genetic pathways (too little sensitivity) or because the aging experiments involved only a few individuals (too much experimental noise). Another previous study compared aging in flies and worms, and reported that there was a common decrease in expression, seen in young adulthood, of genes that encode mitochondrial proteins [19].

In mammals, direct genetic tests of the functional relevance of reduced expression of the electron transport chain pathway on lifespan have not yet been reported. However, in *C. elegans,* reducing the activity of eight genes involved in the electron transport chain using RNAi increased lifespan significantly [35,36]. A gene encoding a subunit of NADH dehydrogenase *(NDUFA10)* is one of the genes showing the largest decrease in expression with age in humans, and its ortholog in worms, *K04G7.4,* was also found to cause one of the largest increases in lifespan using RNAi in C. elegans [36]. Indeed, in these global RNAi screens, the electron transport chain pathway stands out as the pathway showing the largest and most consistent effect on extending lifespan in worms [35]. The genetic results from worms suggest that diminished expression of the electron transport chain pathway in old age in humans may be beneficial, contributing toward extending lifespan.

What types of upstream events might cause a decrease in expression of the electron transport chain pathway with age? Other mitochondrial pathways, such as the mitochondrial ribosome, do not show age regulation similar to the electron transport chain pathway. One potential cause of decreased expression of the electron transport chain pathway is that metabolism may slow in old age, resulting in reduced expression of the energy producing machinery of the cell. Another possibility is that oxidative damage to the proteins in the electron transport chain in the mitochondria may lead to reduced expression of the corresponding genes in the nucleus. The electron transport chain creates free radicals in the process of generating energy that would preferentially damage protein components of the electron transport chain [33].

It seems unlikely that common age regulation of the electron transport chain pathway is directly due to evolutionary conservation. Events in old age are unlikely to have a significant effect on fitness of a population because old animals (such as 3-y-old mice and 80-y-old people) are a small fraction of natural populations (except in recent human history). It could be that the electron transport chain is regulated during aging as an indirect consequence of regulation during development (antagonistic pleiotropy) [37]. Alternatively, age regulation of this pathway may be an unavoidable consequence of aging (e.g., oxidative damage to the electron transport chain in old age may occur in all animals) [33].

It is interesting that the level of age regulation of the electron transport chain is nearly the same in each species, whereas lifespan varies greatly. Compared to humans, mice age 20- to 30-fold and flies age 400-fold more rapidly. Thus, the kinetics of the changes in gene expression for the electron transport chain genes precisely matches the difference in lifespan between species. This suggests that decreased expression of the electron transport chain pathway with age may be particularly informative as a marker of physiological aging.

## Materials and Methods

### Sample collection.

The muscle samples were obtained from patient biopsies collected either during surgery or in an outpatient procedure, and the medical conditions associated with each biopsy are listed in Table S1. For example, the abdominal muscle samples were harvested during surgeries to treat gastrointestinal pathologies. There was no known pathology associated with the abdominal muscle samples themselves, except that they were obtained from patients with various gastrointestinal disorders. In the case of patients with gastrointestinal cancer, the abdominal muscle samples were harvested from regions of the abdomen that were not affected by the cancer. Each muscle sample was immediately frozen in liquid nitrogen and subsequently stored at −80 °C. Finally, we checked each sample by histological staining, and excluded any samples that appeared abnormal or diseased.

### RNA isolation.

Frozen muscle samples were weighed (50–100 mg), cut into small pieces on dry ice, and then placed in 1 ml of TRIzol Reagent (Invitrogen, Carlsbad, California, United States). The tissue was homogenized using a PowerGen700 homogenizer (Fisher Scientific, Pittsburgh, Pennsylvania, United States), and the total RNA was isolated according to the TRIzol Reagent protocol.

### DNA gene chip hybridization.

A standard protocol designed by Affymetrix (Santa Clara, California, United States) for their HG-U133 2.0 Plus high-density oligonucleotide arrays was slightly modified by the Stanford Genome Technology Center (Stanford, California, United States), and all samples were processed in their facility (see Protocol S1). Eight micrograms of total RNA was used to synthesize cRNA for each sample, and 15 μg of cRNA was hybridized to each DNA chip. The samples were processed in random order with respect to age.

### Microarray data normalization and analysis.

We used the DChip program [38] to normalize the data and to generate expression levels for each individual probe set by a perfect-match–only model. All expression data will be publicly available on the Gene Expression Omnibus website upon acceptance. When different probe sets corresponded to the same gene, we averaged the expression levels together. After averaging, we used log_2_-transformed expression values for all subsequent analyses.

### Muscle fiber diameter measurement.

Cross-sections of muscle cryosections were photographed at 200×, and the pictures were either measured digitally (diagnostic muscle biopsy samples, ATPase preparations) or printed (abdominal muscle samples, combined SDH-cytochrome *c* oxidase preparations) and measured by hand. All of the diagnostic muscle biopsies were considered, and 32 of the 81 muscle samples were sufficiently intact for measurement, the remainder being inadequately oriented for cross-sections or too small for meaningful data. Digital analysis consisted of measuring the shortest width through the approximate center of the cell. After calibration with a known length, the diameters were measured and converted to microns using SigmaScan Pro 5.0 software (SPSS Software, Chicago, Illinois, United States). Diameters were tabulated by type I and type II cell types. The counts ranged from approximately 30 cells per type to more than 100 depending on the sample size. Print analysis was by similar methodology. Raw measurements in millimeters were used to calculate the ratio of type II to type I diameters without converting to microns.

### Multiple regression analysis.

To determine the change in expression with age, we used a multiple regression model in which the change in expression with age takes into account the possibility that expression levels might differ in men versus women, or in abdominal muscle versus peripheral muscle. Specifically, we used the following multiple regression model:


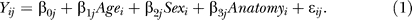


where *Y_ij_* is the expression level of the *j*th probe set for the *i*th sample, *Age_i_* is the age in *y* of the *i*th sample, *Sex_i_* corresponds to the sex of the *i*th sample (0 for male, or 1 for female), *Anatomy_i_* is the anatomic location from which the muscle sample was harvested (0 for abdominal or 1 for peripheral muscle), *ɛ_ij_* represents an error term, *β_1j_* is the change of expression with age, *β_2j_* is the change of expression with sex, *β_3j_* is the change of expression with anatomical origin of sample, and *β_0j_* is the regression intercept. For each gene *j,* we used least-squares to determine all of its coefficients, with our primary interest in the one with respect to age *(β_1j_).* We were interested in genes that show either a positive or negative value for *β_1j_,* indicating either increasing or decreasing expression in old age, respectively.

For human brain, mouse kidney, and *D. melanogaster,* we determined the change in expression with age for each gene using the following multiple regression model:


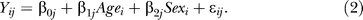


For human kidney, we used the multiple regression model:


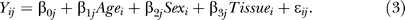


In Equation 3, the tissue term is a binary term scored 0 for cortex and 1 for medulla. For C. elegans data, we used a simple linear regression with age:


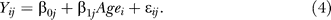


The reviewers suggested two additional methods to show that the age-regulated genes could serve as markers for physiological age. First, we showed that genes regulated by muscle physiology can also predict chronological age. We found genes that were significantly regulated by type II/type I muscle fiber diameter ratio using the multiple regression model:


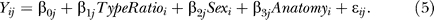


Here, *TypeRatio* is the ratio of type II to type I muscle fiber diameters. We found 585 genes with a statistically significant coefficient for *TypeRatio* using the threshold *p* < 0.01. Of these 585 genes, 114 showed partial correlation with age (absolute value of *r* > 0.2), indicating a significant overlap (*p* < 0.02; hypergeometric distribution) (Table S7). The 92 genes found in the analysis shown in Figure 3 and the 114 genes found in this analysis share a common set of 7 genes, indicating a statistically significant overlap (*p* < 1 × 10^−8^; hypergeometric distribution).

Second, we repeated our age analysis taking into consideration the effect of type II/type I muscle fiber diameter ratio on age regulation. To do this, we used a four-term multiple regression model that includes terms for both age and type II/type I ratio:


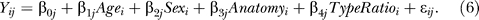


Using Equation 6, we found 543 genes that were regulated by age (*p* < 0.01) and 12,786 genes regulated by type II/type I ratio (*p* < 0.01; Table S8). There are 271 genes shared in common between these two sets of genes, which is a significantly larger number than would be expected by chance (hypergeometric *p* < 1 × 10^−5^; Table S8). We repeated this experiment using a threshold of *p* < 0.001 and found similar enrichment, confirming our results. This analysis shows that the set of genes that are regulated by age is enriched for those that mark the physiology of aging muscle.

### False discovery rate determined by permutation analysis.

We used a permutation analysis to simulate the number of genes that would pass our cutoff by chance (*p* < 0.001). We randomized the age variables of muscle samples 1,000 times while maintaining the sex and anatomy variables with the sample. Equation 1 was used to recalculate regression coefficients and *p* values in every randomization. Theory predicts, and our simulation verifies, that on average about 32 genes pass our threshold (*p* < 0.001) by chance. This result suggests that there are about 13% false positives in our set of 250 age-regulated genes in the muscle. In 95% of the permuted datasets, 107 or fewer genes were significant at the 0.001 level.

### Cluster analysis of pathological and pharmaceutical factors.

To examine whether pathological or pharmaceutical factors were confounding the analysis of age regulation in muscle, we performed unsupervised, average-linkage hierarchical clustering of the 81 muscle samples using the Cluster software [39]. The 81 muscle samples were clustered on the basis of the 250 genes previously determined to be age regulated in human muscle.

### Modified gene set enrichment analysis.

GSEA [40] uses a nonparametric test to decide when the n genes in a group *G* have age coefficients that differ significantly from the N-n genes that are not in *G.* The model is that the n age coefficients in *G* are sampled from a distribution G, while the N-n coefficients not in *G* are sampled from a distribution F. We then test the null hypothesis that F = G. The Kolmogorov-Smirnov test is based on counting how many genes from *G* are in the top K genes of the combined list of age coefficients and comparing it to the number expected when F = G. By letting K vary from 1 to N, the test is sensitive to any alternative F ≠ G. GSEA employs a weighted Kolmogorov-Smirnov test obtained by using a weighted count of genes (with more weight on the extreme ones). In our analysis, we have replaced the weighted Kolmogorov-Smirnov test by a weighted sum, the van der Waerden normal scores test.

The van der Waerden test conforms more closely to our interpretation of what it means for a group *G* of genes to be age related than does the weighted Kolmogorov-Smirnov test. When N is large, then any small group that contains the single most age-related gene is significantly age related by the weighted Kolmogorov-Smirnov test. Such a group displays a genuine statistical significance and comprises strong evidence that F ≠ G, but isn't necessarily biologically increasing or decreasing expression as a mechanistic unit with age. For example, a group of 30 genes with two of the most age-increasing genes and 2 of the most age-decreasing genes could be found to be both an age-increasing group and also an age-decreasing group with significance, even when the other 26 genes are not particularly age related. Here it is clear that F ≠ G, but perhaps it is simply because G has higher variance than F.

To compute the van der Waerden test, we first find the rank r(j) for every gene j ∈ *G*. This rank is the number of the original N genes with an age coefficient smaller than that of gene j. The raw van der Waerden score is


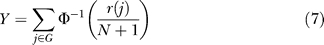


where *Φ* is the standard normal cumulative distribution function. When the N age coefficients are independent with a common continuous distribution F = G, then the distribution of Y is very nearly normally distributed with mean 0 and a variance V*(Y)* close to N-n. We replaced the GSEA enrichment score by the van der Waerden statistic, Z = Y/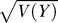 , which is very nearly N*(0,1)* under the null hypothesis. When distribution G is shifted left or right relative to F, then the value of *Z* tends to increase beyond what we would expect from the N*(0,1)* distribution.

### Bootstrap test for significance of GSEA.

It is better to use resampling methods instead of the N*(0,1)* null distribution to assess the significance of the enrichment score Z. The reason is that there are ordinarily correlations among the expression levels of the genes in *G.* When the expression levels of two genes in *G* are correlated, the age coefficients for those genes are correlated as well [41]. It then follows that their ranks are correlated, and this typically increases the variance of Y so that ultimately Z is no longer N*(0,1).* The value of Z can become large either because the genes are age related or because they are correlated with each other. Both may be biologically real, but the second is not an interesting finding, except possibly as confirmation that the group *G* is well constructed.

The original GSEA [40] randomly permutes the labels of two groups being tested while keeping the gene expression data intact. This preserves correlations within the groups so that any significant findings are relative to a null simulation that includes correlations among genes. In many random permutations, one gets a histogram of enrichment scores for age that is centered around zero. If the sample value is far outside the histogram then that enrichment score is statistically significant.

We adopted instead a bootstrap approach. We resampled the data and recomputed enrichment scores, obtaining a histogram roughly centered over the observed enrichment score. If the null value (zero) is far outside the resampled histogram, then the enrichment score is statistically significant. The bootstrap approach also preserves correlations among genes as well as correlations between genes and covariates.

The primary motivation for bootstrapping is the presence of covariates in our problems. Consider for example data with age, sex, and expression variables. If we permute the ages with respect to the expression data and repeat the regression, we have to decide whether the sex variable should be attached to the ages or to the expressions in the random permutation. Attaching sex to the age variables will leave us with simulated data sets in which females express Y chromosome genes as much as males. Because of such artifacts, this is not a suitable null distribution. Attaching a covariate to the expression variables is also problematic. Suppose that one of the covariates is somewhat correlated with age. The effect will be to increase the variance of the originally sampled age coefficient. In permutation samples where the covariate is attached to the expression data, it is resampled independently of age. Such independence reduces the variance of the age coefficient in the permutation data. The consequence is that the permutation-based histogram of age coefficients is then too narrow and false discoveries will result.

In the bootstrap approach we generated 1,000 sample datasets. In each sample dataset we mimicked the sampling process that gave rise to the data by resampling 81 subjects from the population of 81 subjects. The resampling keeps age, expression, and all covariates of any given subject together. Bootstrap sampling mimics the random process that generated the data.

We remark that both bootstrap and permutation sampling of the van der Waerden scores gave rise to Z scores that were nearly normally distributed, but not necessarily N(0,1) (unpublished data). In permutation sampling, the histogram of enrichment scores tended to have means near zero, but several groups had variances larger than 1.0. In bootstrap sampling, the variances often differed from 1 and the means were usually between zero and the original enrichment score.

### Venn and correlation analysis of human muscle, the kidney, and the brain.

The most direct way to compare aging in muscle, the kidney, and the brain is via a Venn analysis: we find which genes attain a stringent significance level for each tissue and judge whether the overlap is statistically significant according to a hypergeometric distribution. We did a pairwise comparison between each tissue to find genes that are aging-regulated in both sets. There are six aging-regulated genes in both the muscle and the kidney (*p* < 0.09, hypergeometric distribution), five aging-regulated genes in both muscle and the brain (*p* < 0.07), and 13 aging-regulated genes in common between the kidney and the brain (*p* < 0.29). There were no genes that were strongly age regulated in all three datasets. The Venn analysis approach is very interpretable but lacks power because it replaces actual measured correlations by a less informative notion of whether they are over a threshold.

A more sensitive comparison can be based on correlating the age coefficients of genes in two tissues. We selected all genes that are age regulated in either of two tissues, plotted the age coefficient of each gene in one tissue versus that gene's coefficient in the other tissue, and computed the Pearson correlation *(r)* of the resulting points (Table S11). We found the strongest overlap in aging between the kidney and the brain (*r* = 0.219), and smaller but positive overlaps in aging between the muscle and the kidney (*r* = 0.103) or the muscle and the brain (*r* = 0.078).

Because the genes are correlated we cannot use textbook formulas to judge the statistical significance of these Pearson scores. To get a *p* value for a Pearson correlation between kidney and muscle, we used 1,000 sets of random genes. The number of genes in each set was the same as the number we used to compute the correlation in Table S11. For each random gene group we computed the Pearson correlation between age coefficients in kidney and muscle. Of the 1,000 samples, there were six in which the random gene group gave rise to a larger Pearson correlation than the one we saw in the real data. This corresponds to a *p* value of 0.006 for kidney–muscle. We similarly found a *p* value of 0.001 for kidney–brain but only 0.058 for muscle–brain. With the possible exception of the kidney–brain pair, the age-related genes have more consistent age coefficients across tissues than randomly selected genes do.

We also ran a bootstrap test of the tissue comparisons. In this test we resampled the microarray data with replacement 1,000 times. Each time we recomputed the correlations between age coefficients for genes in the kidney and muscle. In 1,000 trials we saw 39 in which the sample correlation was less than or equal to zero. After converting to a two-tailed test, this corresponds to a *p* value of 0.078 for kidney–muscle. To save computation, we used the same set of genes in each bootstrap sample instead of making the age-related gene set vary with the sample separately. The *p* value for muscle–brain was 0.07 while that for kidney–brain was 0.001. Based on these individual gene-level analyses, the age-related genes in the kidney and brain tended to be very similar. The muscle–kidney and muscle–brain comparisons were weaker.

### Tests for correlation of tissues within commonly age-regulated gene sets.

To test for the correlation of gene ranks between tissues within those gene sets found to be commonly age regulated in the human, we used a two-tailed Spearman correlation method to first calculate a correlation coefficient for every pairwise combination of tissues (i.e., muscle–kidney, kidney–brain, muscle–brain) for that age-regulated gene set (e.g., extracellular matrix genes). In order to test for the significance of the calculated correlations, we used a permutation-based Monte Carlo method, randomizing the ranks for each gene and tissue in the gene set and recalculating Spearman correlations 1,000 times. We found that most of the correlations between tissues were not significant (Table S12).

## Supporting Information

Figure S1 Age Distribution of Anatomical, Medical, and Pharmaceutical Factors Each row denotes a medical or pharmaceutical factor. Age of patients is shown on the *x*-axis. Sex, biopsy location, and 12 medical factors are shown in the legend. Only hypothyroidism shows any overt association with age.
(253 KB TIF)

Figure S2 Medical and Pharmaceutical Factors do not Affect Age Regulation (A) Coronary artery disease was included as an additional term in Equation 1, and the model was recalculated for the 250 genes that significantly change expression with age. The slope of expression with age (age coefficient) from models with (*y*-axis) and without (*x*-axis) the coronary artery disease term was plotted. If coronary artery disease affected expression, we would expect a large deviation in age coefficient. No significant deviation was seen for any of the 250 age-regulated genes, indicating that coronary artery disease does not adversely affect our study of age regulation.
(B–L) Similar to (A) for 11 other medical factors. (B) Coronary artery disease. (C) Colorectal cancer. (D) End-stage renal disease. (E) Hyperlipidemia. (F) Hypertension. (G) Hypothyroidism. (H) Pancreatic cancer. (I) Prostate cancer. (J) Radiotherapy. (K) Statins. (L) Villous adenoma.
(319 KB TIF)

Figure S3 Cluster Analysis of Medical and Pharmaceutical Factors Samples are clustered on the basis of 250 age-regulated genes in muscle, shown by the top dendrogram. Columns are individual muscle samples, marked by age of the patient. Top seven rows correspond to the expression of the first seven age-regulated genes. The diagram shows anatomical, medical, and pharmaceutical factors for each patient. Each row corresponds to one medical or pharmaceutical factor.
(1.2 MB TIF)

Table S1 Clinical Data (2.1 MB XLS)

Table S2 250 Age-Regulated Genes (*p* < 0.001), Arranged by Slope with Age (59 KB XLS)

Table S3 624 Gene Sets Assayed for Age Regulation (63 KB XLS)

Table S4 Contributing Genes in Gene Sets Age Regulated in Muscle (45 KB XLS)

Table S5 Type II/Type I Muscle Fiber Diameter Ratios of 32 Patients (16 KB XLS)

Table S6 92 Age-Regulated Genes that Predict Type II/Type I Ratio (27 KB XLS)

Table S7 114 Type II/Type I–Regulated Genes that Predict Age (32 KB XLS)

Table S8 Significant Overlap between Sets of Age-Regulated and Type II/Type I–Regulated Genes (14 KB XLS)

Table S9 Contributing Genes in Gene Sets Commonly Age Regulated in Muscle, the Kidney, and the Brain (101 KB XLS)

Table S10 Aging Coefficients of 11,512 Genes in Mouse Kidneys (982 KB XLS)

Table S11 Correlation of Age-Regulated Genes in Three Tissues (15 KB XLS)

Table S12 Spearman Correlations between Tissues for Age-Regulated Gene Sets (15 KB XLS)

## Abbreviations

GSEA: gene set enrichment analysis

## References

[pgen-0020115-b001] Butler RN, Sprott R, Warner H, Bland J, Feuers R (2004). Biomarkers of aging: From primitive organisms to humans. J Gerontol A Biol Sci Med Sci.

[pgen-0020115-b002] Miller RA, Chrisp C, Galecki A (1997). CD4 memory T cell levels predict life span in genetically heterogeneous mice. FASEB J.

[pgen-0020115-b003] Miller RA (2001). Biomarkers of aging: Prediction of longevity by using age-sensitive T-cell subset determinations in a middle-aged, genetically heterogeneous mouse population. J Gerontol A Biol Sci Med Sci.

[pgen-0020115-b004] Krishnamurthy J, Torrice C, Ramsey MR, Kovalev GI, Al-Regaiey K (2004). Ink4a/Arf expression is a biomarker of aging. J Clin Invest.

[pgen-0020115-b005] Rodwell GE, Sonu R, Zahn JM, Lund J, Wilhelmy J (2004). A transcriptional profile of aging in the human kidney. PLoS Biol.

[pgen-0020115-b006] Lu T, Pan Y, Kao SY, Li C, Kohane I (2004). Gene regulation and DNA damage in the ageing human brain. Nature.

[pgen-0020115-b007] Welle S, Brooks AI, Delehanty JM, Needler N, Thornton CA (2003). Gene expression profile of aging in human muscle. Physiol Genomics.

[pgen-0020115-b008] Welle S, Brooks AI, Delehanty JM, Needler N, Bhatt K (2004). Skeletal muscle gene expression profiles in 20–29 year old and 65–71 year old women. Exp Gerontol.

[pgen-0020115-b009] Ly DH, Lockhart DJ, Lerner RA, Schultz PG (2000). Mitotic misregulation and human aging. Science.

[pgen-0020115-b010] Nair KS (2005). Aging muscle. Am J Clin Nutr.

[pgen-0020115-b011] Larsson L, Grimby G, Karlsson J (1979). Muscle strength and speed of movement in relation to age and muscle morphology. J Appl Physiol.

[pgen-0020115-b012] Larsson L, Edstrom L (1986). Effects of age on enzyme-histochemical fibre spectra and contractile properties of fast- and slow-twitch skeletal muscles in the rat. J Neurol Sci.

[pgen-0020115-b013] Fraser HB, Khaitovich P, Plotkin JB, Paabo S, Eisen MB (2005). Aging and gene expression in the primate brain. PLoS Biol.

[pgen-0020115-b014] Lee CK, Klopp RG, Weindruch R, Prolla TA (1999). Gene expression profile of aging and its retardation by caloric restriction. Science.

[pgen-0020115-b015] Lee CK, Weindruch R, Prolla TA (2000). Gene-expression profile of the ageing brain in mice. Nat Genet.

[pgen-0020115-b016] Pletcher SD, Macdonald SJ, Marguerie R, Certa U, Stearns SC (2002). Genome-wide transcript profiles in aging and calorically restricted Drosophila melanogaster. Curr Biol.

[pgen-0020115-b017] Landis GN, Abdueva D, Skvortsov D, Yang J, Rabin BE (2004). Similar gene expression patterns characterize aging and oxidative stress in Drosophila melanogaster. Proc Natl Acad Sci U S A.

[pgen-0020115-b018] Lund J, Tedesco P, Duke K, Wang J, Kim SK (2002). Transcriptional profile of aging in C. elegans. Curr Biol.

[pgen-0020115-b019] McCarroll SA, Murphy CT, Zou S, Pletcher SD, Chin CS (2004). Comparing genomic expression patterns across species identifies shared transcriptional profile in aging. Nat Genet.

[pgen-0020115-b020] Cowie CC, Eberhardt MS, Harris MI, Cowie CC, Stern MP, Boyko EJ, Reiber GE (1995). Sociodemographic characteristics of persons with diabetes. Diabetes in America. 2nd ed.

[pgen-0020115-b021] D'Mello NP, Childress AM, Franklin DS, Kale SP, Pinswasdi C (1994). Cloning and characterization of LAG1, a longevity-assurance gene in yeast. J Biol Chem.

[pgen-0020115-b022] Gulbins E (2003). Regulation of death receptor signaling and apoptosis by ceramide. Pharmacol Res.

[pgen-0020115-b023] Mootha VK, Lindgren CM, Eriksson KF, Subramanian A, Sihag S (2003). PGC-1alpha–responsive genes involved in oxidative phosphorylation are coordinately downregulated in human diabetes. Nat Genet.

[pgen-0020115-b024] Ashburner M, Ball CA, Blake JA, Botstein D, Butler H (2000). Gene ontology: Tool for the unification of biology. The Gene Ontology Consortium. Nat Genet.

[pgen-0020115-b025] Perry SV, Engel AG, Franzini-Armstrong C (2004). Activation of the contractile mechanism by calcium. Myology. 3rd ed.

[pgen-0020115-b026] Maguire TM, Gillian AM, O'Mahony D, Coughlan CM, Dennihan A (1994). A decrease in serum sialyltransferase levels in Alzheimer's disease. Neurobiol Aging.

[pgen-0020115-b027] Maguire TM, Breen KC (1995). A decrease in neural sialyltransferase activity in Alzheimer's disease. Dementia.

[pgen-0020115-b028] Allalouf D, Komlos L, Notmann J, Halbrecht I, Levinsky H (1988). Sialic acid content and sialyltransferase activity in human lymphocytes with advancing age. Mech Ageing Dev.

[pgen-0020115-b029] Colten HR (1992). Tissue-specific regulation of inflammation. J Appl Physiol.

[pgen-0020115-b030] Makrides SC (1983). Protein synthesis and degradation during aging and senescence. Biol Rev Camb Philos Soc.

[pgen-0020115-b031] Jentsch TJ, Maritzen T, Zdebik AA (2005). Chloride channel diseases resulting from impaired transepithelial transport or vesicular function. J Clin Invest.

[pgen-0020115-b032] Mihic SJ, Harris RA (1997). GABA and the GABA_A_ receptor. Neurotransmitter Rev.

[pgen-0020115-b033] Harman D (1956). Aging: A theory based on free radical and radiation chemistry. J Gerontol.

[pgen-0020115-b034] Cheadle C, Vawter MP, Freed WJ, Becker KG (2003). Analysis of microarray data using Z score transformation. J Mol Diagn.

[pgen-0020115-b035] Lee SS, Lee RY, Fraser AG, Kamath RS, Ahringer J (2003). A systematic RNAi screen identifies a critical role for mitochondria in C. elegans longevity. Nat Genet.

[pgen-0020115-b036] Hamilton B, Dong Y, Shindo M, Liu W, Odell I (2005). A systematic RNAi screen for longevity genes in C. elegans. Genes Dev.

[pgen-0020115-b037] Williams G (1957). Pleiotropy, natural selection, and the evolution of senescence. Evolution.

[pgen-0020115-b038] Li C, Wong WH (2001). Model-based analysis of oligonucleotide arrays: Expression index computation and outlier detection. Proc Natl Acad Sci U S A.

[pgen-0020115-b039] Eisen MB, Spellman PT, Brown PO, Botstein D (1998). Cluster analysis and display of genome-wide expression patterns. Proc Natl Acad Sci U S A.

[pgen-0020115-b040] Subramanian A, Tamayo P, Mootha VK, Mukherjee S, Ebert BL (2005). Gene set enrichment analysis: A knowledge-based approach for interpreting genome-wide expression profiles. Proc Natl Acad Sci U S A.

[pgen-0020115-b041] Owen AB (2005). Variance of the number of false discoveries. J Roy Soc Stat B.

